# Biomarkers in Pain

**DOI:** 10.3390/biomedicines11092554

**Published:** 2023-09-18

**Authors:** Anders O. Larsson, Emmanuel Bäckryd, Mats B. Eriksson

**Affiliations:** 1 Department of Medical Sciences, Section of Clinical Chemistry, Uppsala University, 751 85 Uppsala, Sweden; anders.larsson@akademiska.se; 2 Pain and Rehabilitation Center, and Department of Health, Medicine and Caring Sciences, Linköping University, 581 83 Linköping, Sweden; emmanuel.backryd@liu.se; 3 Department of Surgical Sciences, Section of Anaesthesiology and Intensive Care Medicine, Uppsala University, 751 85 Uppsala, Sweden; 4 NOVA Medical School, New University of Lisbon, 1099-085 Lisbon, Portugal

The focus of this Special Issue on Biomedicines is on the value of “Biomarkers in Pain” from a broad perspective.

Pain can be viewed from different perspectives. The pain mechanism can be nociceptive, neuropathic, or nociplastic [1]. Temporally, we usually divide pain into acute and chronic conditions. Acute pain has a protective function and is part of the response to tissue damage. Although causing discomfort, acute pain is often not a major problem as this type of pain usually responds well to pharmacological treatment. In contrast, chronic pain is much more difficult to treat, and, as a rule, treatment options that relieve acute pain cannot alleviate chronic pain; hence, the latter may be regarded as a disease of its own right [2]. The International Association for the Study of Pain defines chronic pain syndromes as persistent or recurrent pain lasting ≥ 3 months [3].

Approximately 20% of the global population suffers from chronic pain [4,5,6,7], although considerably higher proportions have been described, e.g., in the UK, chronic pain affects between one third and one half of the population, which equals almost 28 million adults, a number which may continue to increase further as we face an ageing population [8]. In the United States, 100 million adults suffer from chronic pain, causing an annual economic loss that has been estimated to amount to 600 billion USD [9]. Furthermore, costs associated with pain exceed the costs of heart disease and cancer [10].

Sometimes, acute pain does not resolve. Instead, acute pain may, through hitherto largely still unknown mechanisms, sometimes shift into chronic pain. A problem with both acute and chronic pain is that it is not visible. It may therefore be overlooked or underestimated. Pain is a subjective experience and varies between individuals and different time points. At least for research purposes, it would be desirable to have a more objective way of quantifying pain in addition to a patient’s description of their pain, although important ethical concerns have been raised and are worth pondering [11]. Biomarkers could also provide information of the pathophysiological mechanisms behind pain, allowing for a differentiation between different pain mechanisms and better adaptation of the treatments for each patient. This has triggered the development of potential biomarkers for pain. These biomarkers offer a way of measuring and quantifying pain, reducing the reliance on self-reported pain scales, which can be influenced by psychological and cultural factors. The impact of chronic pain on quality of life is significant, with frequent limitations in ordinary activities of daily life, as well as depression and even suicide [2].

Several previous studies have shown that pain patients’ biofluids, analyzed using modern proteomic methods, exhibit biomarkers that could increase our knowledge on chronic pain and hereby guide its management (e.g., [12,13,14,15]). Such biomarkers may be valuable in order to ensure objectivity in clinical trials when new treatment options are evaluated. In the future, one may expect that biomarkers can help with the diagnosis of various pain conditions and differentiate between different types of pain.

This issue of biomedicines focuses on “Biomarkers in Pain”. It was initially discussed whether “Biomarkers of Pain” would be a more relevant title, but the latter suggestion might imply that a specific biomarker is causative of the agony and not a result of this condition, which could limit and possibly even lead to deceptive conclusions. According to the present state of knowledge, one cannot determine whether the increased levels of biomarkers that are seen in painful conditions are a cause or a consequence. It should rather be remembered that correlation does not imply causation. The relation between biomarkers and pain has previously been validated [14]. Longitudinal studies could possibly help to clarify this issue. Nevertheless, such indicators of pain may be most valuable in order to impart an agonizing process.

This Special Issue of biomedicines highlights several research articles presenting recent advances in the field of Biomarkers in Pain from a broad perspective. Displaying such biomarkers may provide pertinent information about potential new directions for interventions for chronic pain in the research realm. In addition, the review articles presented in this issue may contribute to an increased awareness of biomarkers within a broad context and from a relevant perspective.

In summary, we can expect that pain biomarkers will serve a critical role in improving the diagnosis, treatment, and management of pain conditions. They may offer more objective and precise information based on an individual’s unique genetic and biological makeup, leading to better patient care, drug development, and overall understanding of pain mechanisms.
